# Hair cortisol concentrations in a Spanish sample of healthy adults

**DOI:** 10.1371/journal.pone.0204807

**Published:** 2018-09-28

**Authors:** Maria Angeles Garcia-Leon, Maria Isabel Peralta-Ramirez, Laura Arco-Garcia, Borja Romero-Gonzalez, Rafael A. Caparros-Gonzalez, Noelia Saez-Sanz, Ana Maria Santos-Ruiz, Eva Montero-Lopez, Andres Gonzalez, Raquel Gonzalez-Perez

**Affiliations:** 1 School of Psychology, University of Granada, Granada, Spain; 2 Mind, Brain and Behavior Research Center (CIMCYC), Granada, Spain; 3 Health Psychology, School of Science Health, University of Alicante, Alicante, Spain; 4 Department of Pharmacology, CIBERehd, School of Pharmacy, University of Granada, Granada, Spain; University of Vienna, AUSTRIA

## Abstract

**Background:**

Hair cortisol concentration (HCC), as a novel promising method to retrospectively measure hypothalamic-pituitary-adrenal (HPA) axis activation, is being increasingly studied. We tested the relationships between HCC and a range of possible confounding variables in a Spanish sample of healthy adults and pregnant women.

**Methods:**

The number of healthy adults who participated in the study was 529, being 270 males and 259 females, with a combined mean age of 37.88 years (SD = 15.66). Additionally, a separate sample of 62 pregnant women was also recruited with a mean age of 32.95 (SD = 3.67), and in the first trimester of pregnancy. Each participant was interviewed before the study to obtain sociodemographic and lifestyle variables, and a hair sample was taken from the posterior vertex of the head, cut as close to the scalp as possible. Assuming the average growth rate of head hair is 1 cm per month, a 3-cm segment was analysed, in order to measure the cortisol concentrations from a three-month period. For the pregnant women, hair samples for each trimester of pregnancy were analysed.

**Results:**

The mean hair cortisol concentration was 127.91 (111.52) pg/mg for the general sample. The variables of age, education, employment status, use of hair dyes, use of oral contraceptives, and physical exercise had a significant relation to HCC. When adjusted for further variables, only education and physical exercise remained statistically significant. When including the use of oral contraceptives and only with respect to females, only physical exercise remains statistically significant. For the subsample of pregnant woman, the mean hair cortisol concentration was 334.51 (409.77) pg/mg for the first trimester, 302.18 (270.24) pg/mg for the second trimester, and 331.31 (295.46) pg/mg for the third trimester of pregnancy. None of the assessed confounding variables (age, body mass index, previous miscarriages, employment status, hair dyes, dependent children and physical exercise), except education level, was related to HCC.

**Conclusions:**

In this sample of healthy Spaniards, results suggested an association between HCC and physical exercise and educational level. In pregnant women, the prevalence of HCC was higher than in non-pregnant woman, and was related to educational level. This study emphasises the need to determine the relationship between HCC and confounders such as sociodemographic and lifestyle variables in the general population and specific groups formed by individuals such as pregnant women.

## Introduction

Cortisol is a steroid hormone, or more specifically, a glucocorticoid that is secreted by the adrenal glands. Cortisol affects many bodily systems and plays an important role in bone growth, arterial pressure regulation, immune and nervous system functions, fat, carbohydrate and protein metabolism and, more specifically, in the response to stress [1]. In particular, psychological stress has significant repercussions for physical and psychological health [2]. In some situations, stress can be beneficial. In fact, stress provides an impulse that gives an individual the energy to help others, to skilfully face situations such as passing an exam or getting a job, or to even for arriving on time for a meeting. On the other hand, extreme and prolonged stress severely impacts health and, as aforementioned, can affect the immune, cardiovascular, neuroendocrine, dermatologic, gastrointestinal, and nervous systems [1], as well as triggering mental disorders [3].

Two mechanisms activate the stress response: the sympathetic adrenomedullary system, which secretes catecholamines to induce a rapid response of the cardiovascular system; and the hypothalamic-pituitary-adrenal (HPA) axis. The HPA axis is activated through the secretion of corticotropin-releasing hormone (CRH) by the hypothalamus, which stimulates the pituitary to secrete adrenocorticotropic hormone (ACTH). The ACTH is then transported in the blood to the adrenal cortex, which triggers the secretion of glucocorticoids [4]. One of the most studied glucocorticoids involved in the stress response is cortisol. Currently, the best method of biologically evaluating the response to stress is by measuring HPA-axis activity using cortisol levels. Traditionally, the level of cortisol in saliva has been the most commonly used indicator for measuring cortisol. This method has many advantages: it is non-invasive, therefore, it is less stressful; it is inexpensive; and the data can be collected by non-medical staff in a wide variety of settings. Unfortunately, measuring cortisol levels in saliva, blood (invasive) and urine is only useful for specific, limited periods of time and cannot detect stress longitudinally or retrospectively [5]. Furthermore, these measures are easily influenced by individual and environmental characteristics, such as study procedures [6], the time of day [7], and food consumption [8].

Due to these limitations, researchers have been seeking alternatives that allow for a non-invasive and retrospective assessment of cortisol. In recent years, a new method has been developed for measuring cortisol in humans and animals by extracting cortisol from hair. For years, hair has been used as a substrate for measuring environmental agents, drugs or toxins and even for retrospectively measuring steroid hormones [9, 10], including Hair Cortisol Concentrations (HCC), which were tested for the first time in 2000 [11]. Since then, many studies have begun using this promising method for measuring chronic stress, because, among its numerous advantages, it provides researchers with a window into the recent past of the individual. Hair has a fairly consistent growth rate of approximately 1 cm per month. Therefore, the 1 cm segment closest to the scalp approximates one month of the production of cortisol. The second closest centimetre approximates the previous month’s production, and so on [12]. Hence, by collecting a 3 cm segment of hair, researchers can obtain the accumulated cortisol in hair as an indicator of HPA activation during the three previous months. Researchers are able to retrospectively examine cortisol production during the time period when a stressor was most salient, without needing to take a sample right at that particular time. Moreover, contrary to the method of collecting blood samples, hair sampling is non-invasive and painless. Consequently, there is no risk that the collection itself will stimulate cortisol production. If there would be any particular case of the stimulation of cortisol production caused by the sampling collection, it would have no impact due to delayed cortisol production and scalp-cutting distance. Furthermore, as each centimetre approximates one month’s production of cortisol, intra- and inter-day fluctuations settle. Finally, the ease of use of HCC analysis and the fact that collection does not require trained medical staff are remarkable drivers. Once collected, hair samples can be stored at room temperature, in envelopes or vials, which facilitates its transportation [13].

To date, many studies have applied this method as a biomarker for chronic stress. Since the development and validation of the technique in rhesus macaques (*Macaca mulatta*) [14], hair cortisol research has rapidly increased. In the literature, there are observational or interventional studies [15, 16] that use it as a biomarker for stressful life events [5]; studies on stress in animals [13, 17], studies on emotional states and anxiety disorders and other psychopathological disorders [18, 19], and studies on its relation to other sociodemographic and lifestyle characteristics [20], and others. Accordingly, in the last decade, the method of extracting cortisol from hair has become a promising biomarker for chronic stress and HPA alterations, such as Cushing’s syndrome [21] and Addison’s disease [22]. Moreover, HCC analysis is also a suitable method for known conditions that increase cortisol secretion, which are neither a mental disorder nor a physical/somatic illness, as with pregnancy or endurance athletes [23]. In particular, during pregnancy there is an increase of cortisol production of up to threefold, although the amount of the increase is not clear. This increase appears to be driven by circulating levels of corticotropin releasing hormone of placental origin, which is thought to regulate a placental clock that controls a series of physiological events, including myometrial activation, leading to delivery [24]. In pregnancy, hair cortisol has shown promising results with the finding of relationship with postpartum depression symptoms [25]. Furthermore, relations between newborn hair cortisol and preterm birth and birth weight have been found [26]. The most recent studies have shown how this method facilitates long-term retrospective data-collection on stress, by non-invasive means, which further emphasises its great potential for use in research.

To clarify the effect of possible confounders is relevant for the validity of the HCC methodology. Studies on some of the confounders of HCC are reviewed by Wosu [27] who finds, for example, a complex relationship between age and HCC levels, which seems to be nonlinear. Hence, such a finding highlights the need to develop more studies with a broader age range. In relation to sex, research results are also inconsistent. Research undertaken on lifestyle variables shows no effect of smoking, use of medication, or oral contraceptives, and inconsistent relations with body mass index (BMI), while vigorous physical activity and alcohol intake were positively correlated with HCC. Research on confounders is still in its early stages, and most of studies use of purposive sampling (e.g., caregivers of persons with chronic disease, war veterans, etc.). Therefore, the evidence on the role of possible confounders and the validity of HCC is still scant.

There is a need for research on possible confounders of HCC levels, as well as the possible relation of HCC with a wide range of psychological, sociodemographic and lifestyle variables. Furthermore, it is not clear whether HCC and its relationship with these variables vary along different populations or cultures. To our knowledge, there are no previous studies on HCC using a broad sample from the Spanish population. Therefore, the objective of this research was to study HCC in a sample of healthy Spaniards, to investigate possible confounders of HCC, as well as to study HCC in a sample of pregnant woman.

## Material and methods

### Participants

A sample of healthy Spanish adults, 537 in number, who were primarily from Granada, Jaen, Almeria and Alicante (Spain), participated in this study. The invitation to take part in the study was sent by e-mail and advertisements posted on the notice boards at different public centers. In Granada, participants were recruited at the University (15.3%), employment offices (8.5%), civic centres (9.8%), and day care centres (4%). In Jaen, participants were recruited at the University (10.4%) and civic centres (12.1%). In Almeria, participants were recruited at the University (7%), civic centres (7.8%), and day care centres (2.3%). In Alicante, participants were recruited at the University (15.1%) and civic centres (7.8%). Additionally, a sample of 62 pregnant woman were also recruited when attending at prenatal appointments in 3 public health centres in Granada and Almeria, and in a general hospital in Almeria. Having any physical or physiological illness, using of glucocorticoids or medication known to alter glucocorticoids metabolism and psychiatric disease and being pregnant for women in the general sample were used as exclusion criteria.

After having removed outliers in the cortisol levels, the general sample consisted of 270 males (51%) and 259 females (49%), making a total of 529 participants with a mean age of 37.98 years (SD = 15.66). The separate sample of pregnant women consisted of 62 participants with a mean age of 32.95 (SD = 3.67). The sociodemographic and lifestyle variables are shown in Tables 1 and 2.

**Table 1:** Socio-demographic, lifestyle and hair-characteristic variables from the general sample and their relation to Hair Cortisol Concentrations (HCC) expressed in pg/mg.

Variable		Descriptive	HCC (pg/mg) M (SD)	*df*	Statistics	*p* value	Effect size
Age		37.98 (15.66)			r = -186	.001*	.03
Sex	*Male*	270 (51%)	122.06 (108.02)	527	t = -.43	.66	-
	*Female*	259 (49%)	134.01 (114.94)				
Education	*Primary school*	109 (20.6%)	114.12 (121.67)	3,525	F = 3.38	.01*	.19
	*Secondary school*	58 (11%)	124.99 (93.33)				
	*Vocational education*	78 (14.7%)	131.46 (125.83)				
	*Higher education*	284 (53.7%)	132.82 (106.54)				
Employment status	*Student*	141 (26.7%)	123.88 (110.38)	3,525	F = 13.45	.001*	.07
	*Employed*	264 (49.9%)	137.16 (104.55)				
	*Unemployed*	77 (14.6%)	130.15 (123.7)				
	*Retired*	46 (8.7%)	85.32 (125.27)				
Civil status	*Partner*	243 (45.9%)	125.59 (103.95)	527	t = -.47	.75	-
	*Single*	286 (54.1%)	129.88 (117.71)				
Use of drugs	*Yes*	38 (7.2%)	97.12 (64.78)	527	t = 1.02	.18	-
	*No*	491 (92.8%)	139.61 (139.85)				
Use of contraceptives	*Yes*	48 (18.4%)	175.79 (159.96)	258	t = -2.06	.004*	.45
	*No*	212 (81.5%)	126.85 (99.59)				
Dependent children	*Yes*	183 (34.6%)	146.66 (165.52)	527	t = -.55	.57	-
	*No*	346 (65.4%)	129.64 (117.18)				
Hair	*Natural*	446 (84.3%)	140.78 (143.84)	527	t = 2.20	.04*	.23
	*Dyed*	83 (15.7%)	107.126 (76.95)				
Regular Physical Exercise	*Yes*	243 (45.9%)	137.84 (117.11)	527	t = -2.54	.01*	.11
	*No*	286 (54.1%)	119.47 (106.01)				

Note.

*Significant at the p < .05 level.

**Table 2:** Socio-demographic, lifestyle and hair-characteristic variables from the sample of pregnant women and their relation to Hair Cortisol Concentrations (HCC) expressed in pg/mg.

Variables		Descriptive	1°T HCC (pg/mg) M (SD)	2°T HCC (pg/mg) M (SD)	3°T HCC (pg/mg) M (SD)	*F*	*p* value	*r*^*2*^
Age		32.95 (3.67)				.68	.29	-
BMI		22.75 (2.88)				.44	.44	-
Education	*Secondary education*	18 (29%)	556.46 (577.9)	408.8 (349.39)	304.09 (282.86)	.03*	.03*	.01
	*Higher education*	44 (71%)	243.72 (277.68)	258.56 (220.52)	342.44 (302.94)			
Employment status	*Employed*	48 (77.4%)	366.11 (452.88)	317.84 (295.55)	311.09 (271.81)	.73	.73	-
	*Unemployed*	14 (22.6%)	226.2 (173.38)	248.49 (151.20)	400.59 (368.57)			
Dependent children	*Yes*	26 (41.7%)	324.8 (405.76)	313.86 (312.95)	351.03 (310.35)	.63	.63	-
	*No*	36 (58.3%)	341.53 (418.24)	293.74 (239.06)	317.05 (287.84)			
Previous miscarriages	*Yes*	14 (22.6%)	307.18 (438.61)	359.79 (370.63)	295.37 (275.54)	.65	.65	-
	*No*	48 (77.4%)	342.49 (405.49)	285.37 (235.60)	341.78 (303)			
Hair	*Natural*	34 (54.8%)	408.71 (497.31)	304.4 (266.76)	308.2 (306.65)	.48	.48	-
	*Dyed*	28 (45.2%)	273.41 (315.45)	300.34 (277.06)	350.33 (289.15)			
Regular Physical Exercise	*Yes*	28 (45.2%)	227.47 (254.60)	205.88 (125.2)	347.3 (347.4)	.17	.17	-
	*No*	34 (54.8%)	422.67 (489.5)	381.48 (328.36)	318.13 (249.41)			

Note.

*Significant at the p < .05 level.

The Human Studies Ethics Committee at the University of Granada (Spain) gave approval for this study, which was in accordance with the American Psychological Association’s (APA) Ethical Principles of Psychologists and Code of Conduct [28, 29]. The sample collection was conducted in accordance with the 1975 Helsinki Declaration and its subsequent revisions [30].

### Instruments

#### Semi-structured interview

Age and sex were recorded. Relationship status was categorised as “partner” vs. “single”. The former category included “in a relationship”, “married”, “separated or divorced”, and “widowed” people. The variable dependent children was asked as having one or more children cohabiting and depending economically on the person. Indicators of socioeconomic status included educational attainment and employment status. Educational attainment was divided into “primary school or less”, “secondary school”, “vocational education” and “higher education”. Employment status was divided into “student”, “employed”, “unemployed” and “retired”. In relation to lifestyle measures, physical exercise was framed as “regular physical exercise” vs. “non-regular physical exercise”, as well as consumption of drugs and use of hormonal contraceptives (but not in the pregnant women group). Additionally, the use of hair dyes was registered. Pregnant women were also asked about previous miscarriages, type of conception, and body mass index (BMI).

#### Analysis of HCC

The hair samples consisted of locks of approximately 150 strands of hair taken from the posterior vertex, cut as closely to the scalp as possible. Each sample was then wrapped in aluminium foil to protect it from light and humidity, and stored in an envelope at room temperature. Later, the samples were analysed in the Department of Pharmacology at the University of Granada, Spain.

In our study, we collected 3-cm long hair samples to measure HCC from a 3-month period (assuming an average growth rate of 1 cm per month). After collection, the samples were first washed twice in isopropanol, to remove any cortisol from the outside of the hair shaft that had been deposited from sweat or sebum. After drying, the samples were weighed and ground to a fine powder using a ball mill (Bullet Blender Storm, Swedesboro NJ, USA) to break up the hair's protein matrix and to increase the surface area for extraction. Cortisol from the interior of the hair shaft was extracted into HPLC-grade methanol by incubation of the sample for 72 hours at room temperature in the dark, with constant inversion using a rotator. After incubation, the samples were centrifuged and the supernatant was evaporated until completely dry using a vacuum evaporator (Centrivac, Heraeus, Hanau, Germany). This extract was then reconstituted in 150 uL of phosphate buffered saline (PBS) at pH 8.0. The reconstituted sample was immediately frozen at -20 **°**C for later analysis [22, 31, 32]. Finally, the HCC of each sample was measured using the Cortisol Salivary ELISA kit (Alpco Diagnostics) with phosphate buffered saline (PBS) at pH 8.0. The manufacturer directions for correct usage were provided with the reagent. The cross reactivity, as reported by the manufacturer, is as follows: Prednisolone 13.6%, Corticosterone 7.6%, Deoxycosticosterone 7.2%, Progesterone 7.2%, Cortisone 6.2%, Deoxycortisol 5.6%, Pednisone 5.6%, and Dexamethasone 1.6%. No cross-reaction was detected with DHEAS and Tetrahydrocortisone.

#### Assay variations

The intra-assay variation precision was analysed on three hair samples, which were assayed eight times on the same calibrator curve. The intra-assay coefficients of variance were 5.3% at 2.6 ug/dl, 5.4% at 3.3 ug/dl, and 12.4% at 4.4 ug/dl, respectively. For inter-assay precision three hair samples were analysed on eight separate runs and the coefficients of variance were 13.7% at 2.2 ug/dl, 1% at 3.3 ug/dl, and 10.2% at 4.2 ug/dl, respectively.

### Procedure

The study was organised into two phases. First, all the participants were informed of the study’s objective and the procedure to be followed, before signing the informed consent form. Then, the participants completed the semi-structured interview questionnaire and provided their personal information.

In the second phase, a hair sample was cut from the posterior vertex of the participant’s head. For the general sample, hair was collected in one action, while for the pregnant women, subsample hair was collected in three separate actions over time: at the first trimester (M = 10.55 weeks of gestation; SD = 3.34), the second trimester (M = 24.65 weeks of gestation; SD = 2.34), and the third trimester (M = 34.77 weeks of gestation; SD = 2.07). On average, the entire procedure took approximately twenty minutes in the first collection and five minutes in the second and third collection for pregnant women.

### Statistical analyses

All data were explored and HCC outliers of more than three standard deviations (SD) were excluded [33] (general sample: n = 8; pregnant women: n = 3). Due to the fact that HCCs were not normally distributed, as indicated by the Kolmogorov–Smirnov test, all values were log-transformed for statistical analyses. Untransformed HCCs were, however, reported for descriptive purposes. Descriptive statistics were means and standard deviations for normally distributed variables and relative frequencies for categorical variables. In order to identify all potentially significant determinants of HCC for the general sample, bivariate Pearson correlation coefficients (PCCs) were calculated and simple linear regression, as well as the Student’s *t*-test and univariate ANOVAs with a Bonferroni post-hoc test. For the subsample of pregnant women mixed 2 × 3 analysis of ANOVAs were conducted to check for statistically significant differences between groups, in accordance with each sociodemographic variable. The first factor includes two levels between the independent groups for each sociodemographic variable (secondary vs. higher education, employed vs. unemployed, children vs. no children, miscarriages vs. no miscarriages, natural vs. dyed hair, and physical exercise vs. no physical exercise). The second factor involved repeated-measures within-subjects factors during three trimesters: 1st trimester HCC; 2nd trimester HCC; 3rd trimester HCC. The Greenhouse-Geisser correction was applied in the repeated-measures analyses. When a significant Group x Sampling Time interaction was found, Bonferroni analysis was conducted to determine the trimesters where there were differences between trimesters. Additionally, for all of the confounders with significant effect, effect size was calculated: *r*^*2*^ for normally distributed variables, Cohen’s d and eta-squared for categorical variables. Effect sizes of 0.20 were considered as small, around 0.50 were considered as medium and around 0.80 were considered as large for Cohen’s d. For *r*^*2*^ and eta-squared values of 0.02, 0.13 and 0.25 were considered as small, medium and large respectively [34].

Finally, a multiple linear regression was run, with all relevant confounders (defined as *p* < 0.05) of HCC entered simultaneously. This allowed for mutual adjustment and, as such, enabled us to identify the most relevant confounders of HCC. The statistical significance level was set at alpha = .05. Additionally, percentiles were calculated for the general sample HCC by using the Weighted Average method. Statistical analyses were performed using SPSS 23.0 (IBM Corp., Armonk, N.Y., USA).

## Results

### Sample characteristics

The general sample consisted of 529 participants with a mean age of 37.98 years (*SD* = 15.66), with a broad age range from 18 to 93 years, and the same proportion of males and females (see Table 1). The proportion of single participants and participants in a relationship was also equivalent. The majority of participants had a higher education, were employed at the time of the study, and had no dependent children. A minority (7.2%) of the participants had drug consumption, as well as a small proportion of use of contraceptives in female participants (18.4%). Most of the sample did not use hair dyes and the proportion of participants that practiced regular physical exercise was similar to those who did not practice exercise. The general sample HCC mean was 127.91 (111.52) pg/mg. In order to check the generalizability and comparability of results, sociodemographic data of the study sample was compared to feral data about the general population of Spain. We considered the latest Census of Population and Housing conducted in 2011 by the National Institute of Statistics (INE). The Spanish population has a mean age of 43.14 and is composed by 50.93% of women. In terms of education 19.97% has primary school level, 45.15% has secondary education and 24.28% higher education. That the characteristics of our sample are very similar to those shown by the general population in terms of sex. In relation to educational level, people with higher education and young people were over represented.

The sample of pregnant women consisted of 62 females in the first trimester of pregnancy with a mean age of 32.95 (3.67), range: 25–41 years (see Table 2). Most of them had a higher education and were employed. All of them were in a relationship and almost half (41.7%) had dependent children. All of them had a natural conception and 22.6% had previous miscarriages. None of them had drug consumption and nearly half practiced regular physical exercise. The use hair dyes was practised by 42%. The HCC mean for the first trimester was 334.51 (409.77) pg/mg, for the second trimester was 302.18 (270.24) pg/mg, and for the third trimester was 331.31 (295.46) pg/mg.

### Sociodemographic and lifestyle variables and their relation to HCC

**Fig 1:**
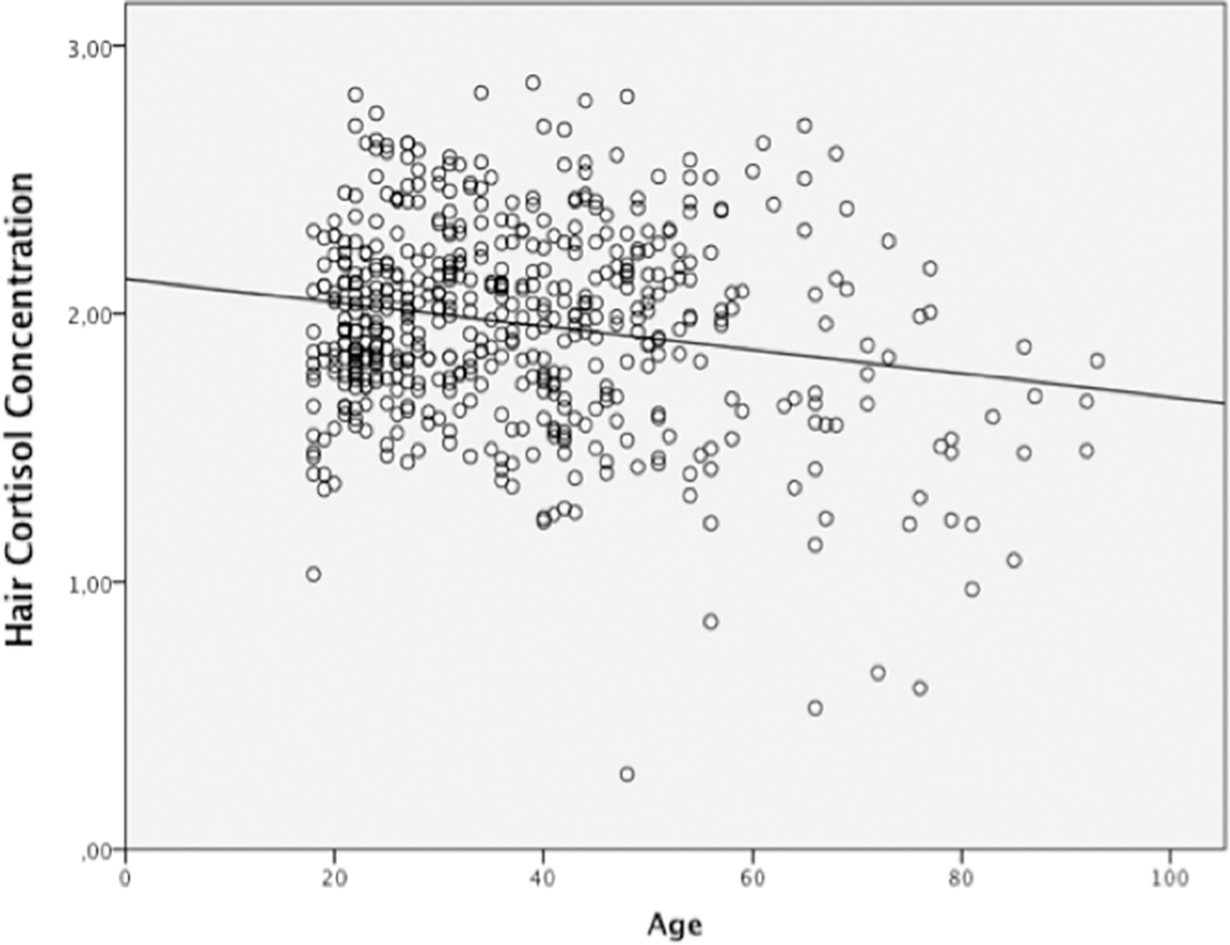
Age plotted against hair cortisol concentrations. The line shows unadjusted correlation. Linear regression adjusted for age: log-transformed Hair Cortisol Concentrations. *r*^*2*^ = .03, *p* < .001.

For the general sample, as can be seen in Table 1, age was significantly associated with HCC with a negative correlation and a low effect size (*r*^*2*^ = .03), see Fig 1. Participants with different levels of education showed statistically significant differences in HCC, with higher levels of HCC for the participants with a higher education than participants with a primary education, according to post-hoc analysis. However, eta squared shows a low effect size for education of .19. Employment status was also significantly associated with HCC; post-hoc analysis showed that retired participants had lower levels of HCC than all others participants, but eta squared show a low effect size for employment status of .07. The Student’s *t*-test show statistically significant differences for the use of contraceptives, hair dyes and regular physical exercise; with higher levels of HCC for participants with natural hair, females using contraceptives, and participants who practiced regular physical exercise. Cohen’s d showed a low effect size for hair dyes (d = .25) and physical exercise (d = .11) and a medium effect size for the use of contraceptives (d = .45).

For the subsample of pregnant women, bivariate Pearson correlations showed no significant relation between HCC during the three trimesters and age and BMI. The mixed 2 × 3 ANOVA analysis with repeated measures shows interaction only between groups of participants with a secondary education level, and participants with a university level education. Bonferroni analysis showed significant differences in the first trimester of pregnancy (*p* = .01) with an effect size of .68 (Cohen’s d), but not in the second (*p* = .08) and third trimesters (*p* = .37). Pregnant women with a secondary level of education showed higher levels of HCC than pregnant women with university studies. Means and standard deviations are presented in Table 2.

### Multiple predictors of HCC

All previously identified confounders with a significant effect on HCC (age, education level, employment status, use of hair dyes and regular physical exercise) were entered simultaneously into one regression model. Use of contraceptives, valid only for females, was included in a regression analysis, along with the rest of the variables and run only for female participants. Bivariate Pearson correlation and Spearman correlation below .80 indicated no collinearity between variables [33]. Only education level and regular physical exercise remained predictors of HCC for the general sample (see Table 3 for the complete model). Using only these significant variables in a regression analysis resulted in an explained variance of the regression model of .03 (adjusted *r*^*2*^). For females, when introducing the use of contraceptives along with the other variables, only regular physical exercise remains a predictor of HCC. The regression analysis for regular physical exercise resulted in an explained variance of the regression model of .01 (adjusted *r*^*2*^).

**Table 3:** Mutually adjusted confounders of log-transformed hair cortisol concentration; multiple linear regression.

	β	*p* value	*r*^*2*^	*Model r*^*2*^	*r*^*2*^ adj
Age	-.077	.23		.059	.05
Education	.105	.03*	.019		
Employment status	.059	.38			
Hair dye	.082	.06			
Physical exercise	.104	.01*	.012		

Note.

*Significant at the p < .05 level.

Model fit: F(5,523) = 6.53; *p* < .001.

### HCC percentiles for the general sample

Considering the large sample from the Spanish population of the present study, we have elaborate percentiles in order to show the data distribution of HCC. Results did not show strong relations between HCC and any of the sociodemographic or lifestyle variables, hence percentiles were elaborated with no grouping. Percentiles were calculated using the Weighted Average method and are shown in Table 4.

**Table 4:** Percentiles for HCC in the general sample.

Percentile	HCC pg/mg
5	24.4
10	31.3
15	38.7
20	45.9
25	54.8
30	60.5
35	68.4
40	14.2
45	85.6
50	95.4
55	105.0
60	118.2
65	127.4
70	139.7
75	156.2
80	148.7
85	221.8
90	269.5
95	361.2

## Discussion

In recent years, the measurement of HCC has been on the rise, because of its significance in evaluating chronic stress and other associated disorders, such as depression or post-traumatic stress. Furthermore, HCC is useful for evaluating disorders that entail inadequate HPA response, such as Cushing’s disease, Addison’s disease, or various autoimmune diseases. As this method becomes increasingly more prevalent, a deeper understanding about its relationship with other confounder variables is needed, as well as, information about HCC in different groups and populations. Therefore, the objective of this research was to study HCC in a sample of healthy Spaniards, to research on possible confounders of HCC, as well as to study HCC in a sample of pregnant woman. Results have shown a significant relation between HCC and age, education, employment status, physical exercise, and use of hair dyes and contraceptives. However, in the adjusted model, only education and physical exercised remained as predictors. Pregnant women showed higher levels of HCC than non-pregnant women during the three trimesters of pregnancy, and their HCC was related to education level in the first trimester.

The effect of age has been studied in several studies, finding different relations with varying hair cortisol levels. According to the meta-analysis of Stalder [23], age was found to be positively related to HCC, although only in a correlation-based analysis. In this line, Staufenbiel [18] finds a positive lineal relation between age and cortisol in a sample of 760 participants (16 to 65 years of age), while Dettenborn [35] finds a quadratic relation with a U-shaped relation, in a sample of 360 participants with ages from 1 to 91 years. In our sample, results show a negative linear relation with lower HCC in older ages. An explanation for this discrepancy may be the sample characteristics, while our sample is constituted by healthy adults, other studies do not exclude illness, although they do analyse the effect of some illness on HCC. Staufenbiel [18] finds higher HCC in participants with diabetes mellitus, the incidence of which increases with age. Therefore, our results may show a decrease in HCC with age, due to the removal of some illness effect.

In relation to the sex of the participants, our results show no statistical differences between women and men. In accordance with this, previous investigations reveal no sex differences in hair cortisol levels [36, 37], while other researchers suggest lower HCC in women than in men [35, 36]. Therefore, further studies are needed to clarify the relation of HCC with confounders, such as age and sex. With respect to other sociodemographic variables, our results show no statistical differences in civil status, dependent children, and the use of drugs. These results are similar to those found by Dettenborn [35], Staufenbiel [36], and Feller [38]. Related to education, our results show higher levels of HCC in participants with a university level of education than, participants with a primary level of education or less. Previous research found no effect of education on HCC [36, 39]. In contrast, Boesch [40] finds a negative relation between HCC and education in young men who were occupied with military training. In this line, retired participants showed lower levels of HCC than employed, unemployed and student participants. There are no previous works, to our knowledge, that study employment status in a wide age range, therefore, there are no studies considering these four categories (employed, unemployed, students and retired) and their relation with HCC. Only Feller [38] finds higher HCC, related to retired and unemployed status in older adults, although this effect disappeared when adjusting for further confounders. Considering retired status to be more prevalent in older ages, our results also show lower levels of HCC in older ages. Although the effects of these variables disappear when adjusting for further confounders, our sample constitutes a healthy sample, and this fact may explain the discrepancies within the results of other works, due to the removal of some illness effect.

In the review by Wosu [27], vigorous physical exercise seems to be related to higher HCC levels, and similar results are disclosed by Gerber [41] in comparing moderate vs. vigorous physical exercise in university students. In our study, “regular physical exercise” reported by participants was positively correlated with HCC levels. Considering the wide age range of our study, this may be attributable to the different impacts of physical activity, depending on the age of the individual. Regarding the effect of hormonal contraceptives in HCC, our results show higher levels of HCC in females using hormonal contraceptives. Although some studies find no effect related to the use of contraceptives [23, 27], others studies find the same relation [35, 36, 39] that appears in the results of the present study. According to Burke [42], combined oral contraceptives increase cortisol production, but this depends on the dose of oestrogen and, when considering the complex effect of estrogen on the hepatic metabolism of steroids, it is not surprising that results showing that HCC can be dependent on oral contraceptives use are mixed. The use of hair dyes and their effect on HCC also has mixed results. In our study, natural hair was related to lower HCC according to previous studies [31, 43], while other studies show no effect of hair dyes [20, 39, 44]. Therefore, further research is needed to clarify the effects of contraceptives and hair dyes on HCC.

HPA activation during pregnancy has a relevant effect in perinatal outcomes, furthermore, pregnancy is related to a natural increase of HCC levels. Previous works have shown an increase of HCC during pregnancy [24, 45], while others has shown lower levels in the second trimester related to postpartum depression symptoms [25], however our results did not show statistically significant differences between trimesters, this highlight the need for more research to clarify how HCC vary during pregnancy in relation to others variables. In relation to sociodemographic variables, our results show higher levels of HCC in pregnant women with lower levels of education (secondary level or less) than women with higher levels of education (university education). To our knowledge, only the study by Braig [46] assesses the relation between HCC and educational level and finds the same results, whereby, pregnant women with university studies level had lower levels of HCC than pregnant women with lower levels of education. However, we do not find interactions of HCC with age, BMI, previous miscarriages, employment status, physical exercise, use of hair dyes or dependent children. In relation to BMI, Braig [46] finds higher levels of HCC only in obese participants, while Scharlau [47] finds no relation between HCC and BMI. In relation to employment status, Braig [46] finds higher levels of HCC in multiple jobholding individuals, but not in the unemployed or in women with only one form of employment. Although we do not consider the variable of multiple jobholding, in this line our results also suggest no differences between employed and unemployed women, as suggested by our results in relation to employment and unemployment. In relation to age, previous studies [46, 47] also show no effect of age on HCC.

Some limitations of the present study are noted here. First, young participants with a high educational level are over-represented. Consideration of having any illness as an exclusion criteria, permitted us to exclude the confounder effect of many health problems, but also to reduce the number of possible participants in older ages. Moreover, while there are controversial results related to the gender of the sample and its relation with HCC, to our knowledge, there are no investigations setting differences in HCC across different levels of education or employment status by using a sample with a wide range of ages. In relation to education, only Boesch [40] finds significant differences in HCC in a sample of young males undertaking military training, however, the characteristics of this sample do not permit comparisons with our results. Another limitation of our research has been the non-inclusion of other variables which may have an effect in HCC, for example, smoking, body mass index, hair washing frequency or heat treatments. Research using these variables shows inconclusive results [23, 27], hence, it would be necessary for further research in assessing the relation between these variables and HCC. We include BMI in the sample of pregnant women, but not in the general sample, however, many recent studies have shown higher levels of HCC in obese participants, which may constitute a potential bias to HCC means in our results. On the other hand, in relation to self-reported measures of stress, we do not include any assessment of perceived stress or life events due to the fact that previous research has shown no relation between HCC and self-reported measures of stress, both in the general population and in pregnant women [23]. Although a relation between HCC and ongoing chronic stress has been found [23], we do not control for this variable, which may also constitute a potential bias to HCC means in our results. Related to the method of extraction, different methods or different ELISA kits show different results [22], which may limit any comparison between studies. However, a recent study investigating the inter-laboratory consistency in determining HCC using different methods, has found a high correlation between methods and laboratories when analysing a common batch of hair [48]. Moreover, further investigations are needed, in order to permit comparisons among different methods and laboratories.

Despite the limitations, to our knowledge, this is the first instance of a research with a large sample and a wide age range from the general population of Spain, in studying HPA activation via HCC means and its relation with sociodemographic variables, as well as using a sample of pregnant women. Considering hair cortisol as an important factor that is associated with endocrine functioning, as well as with a health risk for the general population, and specially during pregnancy, such a biomarker would also potentially help facilitate the earlier detection of individuals who are most at risk for deleterious health outcomes, and help to develop preventive methods to mitigate stress. Additionally, normative scores will help to better understand conditions associated with HPA functioning, such as Cushing syndrome, depression, or Post-traumatic stress disorder (PTSD) [49, 50].

## Conclusions

Research using the HCC methodology is still in its early stages. Therefore, the body of evidence is still little or non-existent and also it is inconclusive. In our sample of healthy Spaniards, HCC decreases with age. We also find an interaction with educational level, employment status, use of contraceptives, use of hair dyes, and physical exercise. In our sample of pregnant women, we find higher levels of HCC than in non-pregnant women during the three trimesters of pregnancy. Only during the first trimester did we find a relation with education level. This study emphasises the need to determine the relationship between HCC and confounders such as sociodemographic and lifestyle variables in the general population and specific groups formed by individuals such as pregnant women. HCC has the potential of becoming a valuable tool in diagnosing and controlling the progression of HPA diseases and conditions which have an effect on HPA functioning. Further research is needed to clarify the value of HPA in clinical practice and research, and also in order to improve the methodology, its application and the interpretation the research results.
