# Correction: Hair cortisol concentrations in a Spanish sample of healthy adults

**DOI:** 10.1371/journal.pone.0221883

**Published:** 2019-08-26

**Authors:** Maria Angeles Garcia-Leon, Maria Isabel Peralta-Ramirez, Laura Arco-Garcia, Borja Romero-Gonzalez, Rafael A. Caparros-Gonzalez, Noelia Saez-Sanz, Ana Maria Santos-Ruiz, Eva Montero-Lopez, Andres Gonzalez, Raquel Gonzalez-Perez

In Table 4, the HCC pg/mg value for the 40^th^ percentile should be 74.2 and the HCC pg/mg value for the 80^th^ percentile should be 184.7. Please see the correct Table 4 here.

**Table 4:** Percentiles for HCC in the general sample.

Percentile	HCC pg/mg
5	24,4
10	31,3
15	38,7
20	45,9
25	54,8
30	60,5
35	68,4
40	74,2
45	85,6
50	95,4
55	105
60	118,2
65	127,4
70	139,7
75	156,2
80	184,7
85	221,8
90	269,5
95	361,2
